# CREATING THE CT GERONTOLOGY CONSORTIUM TO BRING FACULTY AND STUDENTS TOGETHER TO PROMOTE GERONTOLOGICAL EDUCATION

**DOI:** 10.1093/geroni/igz038.1971

**Published:** 2019-11-08

**Authors:** Christina Barmon, Claudia E Oakes, Donna Fedus, Erica Michalowski, Andrea June, Carrie Andreoletti

**Affiliations:** 1 Central Connecticut State University, New Britain, Connecticut, United States; 2 University of Hartford, West Hartford, Connecticut, United States; 3 Borrow My Glasses, Madison, Connecticut, United States; 4 AARP CT, Hartford, Connecticut, United States

## Abstract

Although commonplace for faculty across universities to collaborate on research, it is less common to bring faculty and students from different institutions together for pedagogical purposes. In this poster, we describe the development of the Connecticut Gerontology Consortium, comprised of gerontology instructors at several academic institutions. We created this consortium in order to 1) develop a support network for faculty interested in teaching about aging, and 2) to support collaboration on efforts to promote creative, interdisciplinary student experiences that foster a passion for gerontology. In addition, we describe one endeavor, a student project, from the planning stages to post-project reflections. The intercollegiate and interdisciplinary “Careers in Aging” project brought together 62 students from four academic institutions with a broad range of experiences and interests. Students were assigned to groups from different schools to investigate careers in the aging network in Connecticut. Each student group interviewed a professional, delivered a presentation about their career path and day-to-day experiences, and created an informational brochure about the profession. Students indicated that they valued the experience of learning about the aging network and engaging with other students who shared an interest in gerontology. However, they cited challenges in communicating with students from other classes and at other institutions. This project has the potential to build networking opportunities for students as well as strengthening the community of gerontology professionals. The presentation will include lessons learned and how the consortium plans to move forward.

